# Psychological stress in aged female mice causes acute hypophagia independent of central serotonin 2C receptor activation

**DOI:** 10.1371/journal.pone.0187937

**Published:** 2017-11-10

**Authors:** Chinami Matsumoto, Chihiro Yamada, Chiharu Sadakane, Miwa Nahata, Tomohisa Hattori, Hiroshi Takeda

**Affiliations:** 1 Tsumura Research Laboratories, Tsumura & Co., Ibaraki, Japan; 2 Pathophysiology and Therapeutics, Faculty of Pharmaceutical Sciences, Hokkaido University, Sapporo, Hokkaido, Japan; 3 Hokkaido University Hospital Gastroenterological Medicine, Sapporo, Hokkaido, Japan; Kent State University, UNITED STATES

## Abstract

Sex differences exist in the activation of the hypothalamic–pituitary–adrenal axis following exposure to stress, and the stress response is further affected by aging. This study was conducted to elucidate the mechanism of hypophagia in aged female mice exposed to stress. Immediately after a stress load, aged female mice exhibited acute hypophagia and a rise in plasma corticosterone levels. The administration of a serotonin 2C receptor (5-HT_2C_R) antagonist suppressed plasma corticosterone but did not affect the reduction in food intake. In contrast, an endogenous ghrelin enhancer, rikkunshito (RKT), significantly inhibited the reduction in food intake. An increase in peripheral acylated ghrelin levels during fasting, which occurs in young mice, was not observed in aged female mice. Moreover, in these mice, significantly increased levels of ghrelin and gastric preproghrelin mRNA expression were observed in the fed status. Moreover, plasma ghrelin levels were elevated by RKT and not by the 5-HT_2C_R antagonist. In female mice, the hypothalamic non-edited (INI) and partially edited mRNA 5-HT_2C_R isoforms (VNV, VNI, VSV or VSI) decreased with age, while in male mice, the editing isoform was unchanged by aging or stress. Estrogen receptor α (ERα)-positive cell counts in the arcuate nucleus of young male mice exposed to stress and control aged male mice were increased compared with those in young control mice. In aged male mice exposed to stress, the number of ERα-expressing cells in the paraventricular nucleus were significantly increased compared with those in aged control mice; in female mice, there was no increase in the number of ERα-positive cells. Hypophagia in aged female mice exposed to stress may be independent of 5-HT_2C_R activation. It seems likely that the mechanisms may be caused by sex dependent, differential regulation in 5-HT_2C_R mRNA expression, peripheral acylated ghrelin secretion and/or hypothalamic ERα expression.

## Introduction

Adverse life events are associated with the onset and persistence of depression [1], and depression is characterized by feeding abnormalities. In particular, the combination of depression and anorexia may influence morbidity and progressive physical disability among the elderly [2, 3].

Sex differences exist in the activation of the hypothalamic–pituitary–adrenal (HPA) axis following exposure to stress [4–6]. The incidences of major depression [7] and anorexia nervosa [8] are higher in women than in men. In young rodents, the activation of the HPA axis due to stress is greater in females than in males [9] and is regulated by sex hormones [4, 5]. Aging is one of the major factors that affects appetite. Age-related anorexia is more pronounced in males [10, 11]. Furthermore, late-life depression in males is a risk factor for mortality [12, 13]. In addition, aging may even interfere with phenotypes that cause sex differences such as stress. Compared with aged male mice, aged female mice exposed to novelty stress have a milder reduction in food intake after stress, however, the mechanism underlying this observation is unclear [14].

Corticotropin-releasing factor (CRF) and serotonin (5-HT) play important roles in stress responses and the regulation of feeding behavior [15, 16]. The activation of CRF neurons during stress suppresses feeding behavior [17, 18]. CRF production and neuron activation may also be mediated by 5-HT. The serotonin 2C receptor (5-HT_2C_R), localized on CRF neurons of the hypothalamus, stimulates anxiety [19–21] and negatively regulates food intake [22–25]. The gene expression of 5-HT_2C_R is increased in the paraventricular nucleus (PVN) of aged male mice [26]. Neural hyperactivity by stimulation of 5-HT_2C_R in the PVN or amygdala due to exposure to stress suppresses the secretion of the peripheral orexigenic hormone ghrelin, leading to a decrease in food intake. The involvement of 5-HT_2C_R activation after a stress response in aged female mice is unknown. Excessive, central activation of the 5-HT_2C_R in aged male mice is caused by increased gene expression [26]. Furthermore, changes in the editing of the 5-HT_2C_R influence the affinity or signal transduction of the receptor and are involved in neuropsychiatric diseases [27]. Therefore, we examined the influence of aging and sex on changes in the gene expression of the pre-edited 5-HT_2C_R.

Estrogen regulates food intake, and an ovariectomy increases both and weight gain [4, 28]. The estrogen receptor α (ERα) is widely expressed in the central area of the brain, including the hypothalamus, and negatively controls feeding [29]. The activation of the 5-HT_2C_R promotes the synthesis of ERα in the dorsal raphe region [30] and hypothalamus [31]. We found that hypophagia in aged male mice exposed to stress may be mediated by the interaction of ERα with 5-HT_2C_R activation [14]. However, the effects of sex differences and aging on ERα expression in the hypothalamus in stress exposed mice are not well understood.

This study investigated the hypothesis that the reduction in food intake after exposure to stress in aged female mice is mediated by an independent decrease in ghrelin secretion by the activation of the 5-HT_2C_R, leading to ERα expression. First, we identified the effects of 5-HT_2C_R antagonists or an endogenous ghrelin enhancer [32–34] on feeding behavior, exploring the role of the 5-HT_2C_R or ghrelin in aged female mice exposed to novelty stress. Next, we determined the levels of plasma ghrelin and the expression of hypothalamic ghrelin-related genes during the suppression of feeding in aged female mice. Finally, we conducted a study of the expression of the edited 5-HT_2C_R gene and ERα-positive cells in the hypothalamus during the novelty stress-induced suppression of feeding behavior.

## Materials and methods

### Animals

Male and female C57BL/6 mice aged 6 weeks and over 79 weeks were purchased from Charles River Laboratories, (Tokyo, Japan). We used elderly mice before the onset of aging cachexia that weighed 45 g or less with no abrupt changes in body weight and no apparent injuries. In addition, no changes in voluntary movement, such as sedation, were used. Therefore, the number of mice in the control and experimental groups did not match. Before the experiments, five mice per cage were acclimated to a temperature- and humidity-controlled room with a 12 h light/dark cycle (lights on at 07:00AM) with *ad libitum* access to food and water. This study was approved by and conducted according to the guidelines of the experimental animal ethics committees of Tsumura & Co. (Ibaraki, Japan; permit no: 09–126, 09–151, 11–135)

### Effects of exposure to novelty stress on food intake, plasma corticosterone levels, and plasma ghrelin levels in young and aged mice

The novelty-induced hypophagia test evaluates the degree of anxiety or depression based on the suppression of food intake after exposure to a novel environment [35]. Tests were conducted as previously described [14, 26]. Mice were housed in groups [five mice/cage; cage size (width × depth × height) = 230 × 310 × 155 mm] for seven days before the experiment; cages were not changed until the experiment was completed. To induce novelty stress, some of the group-housed mice (five mice/cage) were transferred to separate cages (one mouse/cage; cage size (width × depth × height) = 136 × 208 × 115 mm) under *ad libitum* access to food and water or after 18 h of a fasted condition. The remaining group-housed mice served as controls. Food intake was determined at various time intervals, at 3 h after exposure to novelty stress, the mice were sacrificed by decapitation without anesthesia, and blood was collected from the different animal groups. Aprotinin and EDTA-2Na were used during blood collection, and the collected blood was centrifuged immediately to collect the plasma. The dead animals were exsanguinated after blood collection. To determine blood ghrelin levels, 10% HCl (1 N) was added to the plasma and the levels of acylated ghrelin and desacylated ghrelin were measured in the plasma. Plasma corticosterone and ghrelin levels were determined using the corticosterone enzyme immunoassay kit (Enzo Life Sciences, Plymouth Meeting, PA, USA) and the Active Ghrelin ELISA Kit/ Desacyl-Ghrelin ELISA Kit (LSI Medience Corporation, Tokyo, Japan), respectively. Samples were collected between 1:00 PM and 3:00 PM to avoid diurnal variations.

First, to clarify the sex difference in aged mice, food intake was evaluated at 6 and 24 h after exposure to novelty stress. To evaluate food intake per mouse, mice housed individually for seven days before the experiment with *ad libitum* access to food and water and in the fed condition served as the control group. We confirmed that there was no difference in the mean food intake between these individually housed mice and the group-housed mice, calculated by dividing the food intake for each cage by the number of mice in each cage [26]. We also confirmed that plasma corticosterone levels in individually housed mice were similar to those in group-housed mice [36], as previously reported [37].

### Effects of test drugs on food intake and plasma corticosterone or ghrelin levels

To investigate the effects of 5-HT_2C_R antagonists on food intake in aged mice, the selective 5-HT_2C_R antagonists SB242084 (Tocris Bioscience, Glasgow, UK, 6 mg/kg, [23, 38]) and rikkunshito (RKT; Tsumura & Co., Tokyo, Japan; 1,000 mg/kg, [32]) were suspended in distilled water and orally administered by gavage (10 mL/kg) to the 18 h-fasted mice immediately after they were exposed to novelty stress. Moreover, the cumulative food intake was determined at 1 h and 3 h after exposure to the novelty stress. Acute hypophagia occurs at these time points due to the stress load, even in aged female mice. We previously confirmed that the food intake between control and stressed female mice at 6 h or 24 h is unchanged and that the oral administration of SB242084 (6 mg/kg) and RKT (1,000 mg/kg) to these mice has no effect [14]. To clarify the role of the 5-HT_2C_R on plasma ghrelin levels, the oral administration of SB242084 (6 mg/kg) or RKT (1,000 mg/kg) to 18 h-fasted, aged male mice was performed immediately after exposure to novelty stress and their blood was collected 3 h after this exposure to determine their plasma ghrelin levels.

### Total RNA extraction and reverse transcription polymerase chain reaction (RT-PCR)

The hypothalamus or stomach was rapidly removed from each fed or fasted mouse and immediately frozen in a tube maintained on dry ice. Isolated tissue homogenization and total RNA extraction were performed using the RNeasy Universal Tissue kit (Qiagen, Valencia, CA, USA). Diluted total RNA (100 ng/μL) was incubated at 70°C for 5 min and then cooled on ice. Total RNA (1,000 ng) was reverse transcribed using the TaqMan Reverse Transcription Reagents kit (Applied Biosystems, Foster City, CA, USA). Furthermore, quantitative PCR assays were performed on a Prism 7900HT Sequence Detection System (Applied Biosystems) using the TaqMan Gene Expression Master Mix (Applied Biosystems), TaqMan gene-specific primer/probes (*Rps29*, Mm02342448_gH; Npy, Mm00445771_m1; Agrp, Mm00475829_g1; Pomc, Mm00435874_m1; Ghrl, Mm00445450_m1) and 5-HT_2C_R editing primer/probes (Htr2c_INI, tcaactgcgtccatcatgcacctctgcgccatatcgctggaccggtatgtagcaatacgtaatcctattgagcatagccg; Htr2c_VNV, tcaactgcgtccatcatgcacctctgcgccatatcgctggaccggtatgtagcagtgcgtaatcctgttg; Htr2c_VNI, tcaactgcgtccatcatgcacctctgcgccatatcgctggaccggtatgtagcagtgcgtaatcctattgag; Htr2c_VSV, tcaactgcgtccatcatgcacctctgcgccatatcgctggaccggtatgtagcagtgcgtagtcctgt; Htr2c_VSI, tcaactgcgtccatcatgcacctctgcgccatatcgctggaccggtatgtagcagtgcgtagtcctattg [39–41]). The mRNA expression of the gene of interest versus that of a housekeeping gene (ribosomal protein S29) was calculated using the ΔΔCt method.

### Immunohistochemistry

The hypothalami from young and aged male and female mice were rapidly removed while being cooled by ice 6 h after stress exposure and were perfusion-fixed with formaldehyde. For the first immunohistochemistry analysis, tissue sections were de-paraffinized with xylene and rehydrated through an ethanol series and Tris-buffered saline. Antigen retrieval was performed by microwave treatment, with citrate buffer, pH 6.0. Endogenous peroxidase was blocked with 0.3% H_2_O_2_ in methanol for 30 min, followed by incubation with Protein Block (Genostaff, Tokyo, Japan) and an avidin/biotin blocking kit (Vector). The sections were incubated with anti-ERα rabbit polyclonal antibody (Santa Cruz) at 4°C overnight. They were incubated with biotin-conjugated goat anti-rabbit Ig (Dako, Tokyo, Japan) for 30 min at RT, followed by the addition of peroxidaseconjugated streptavidin (Nichirei, Tokyo, Japan) for 5 min. Peroxidase activity was visualized by diaminobenzidine and then each section was washed with PBS.

### Statistical analyses

Two-way factorial analysis of variance followed by the Tukey–Kramer post hoc test was used for the comparison between young and aged mice. Data from the experiments on aged mice were analyzed using Student’s *t*-test and one-way analysis of variance, followed by Dunnett’s test or Steel’s test. Data are presented as the mean ± standard error for each group, and *p* < 0.05 was considered statistically significant.

## Results

### Effects of the 5-HT_2C_R antagonists, SB242084 and RKT, on the decrease in cumulative food intake and plasma corticosterone levels in aged female mice exposed to novelty stress

**Fig 1:**
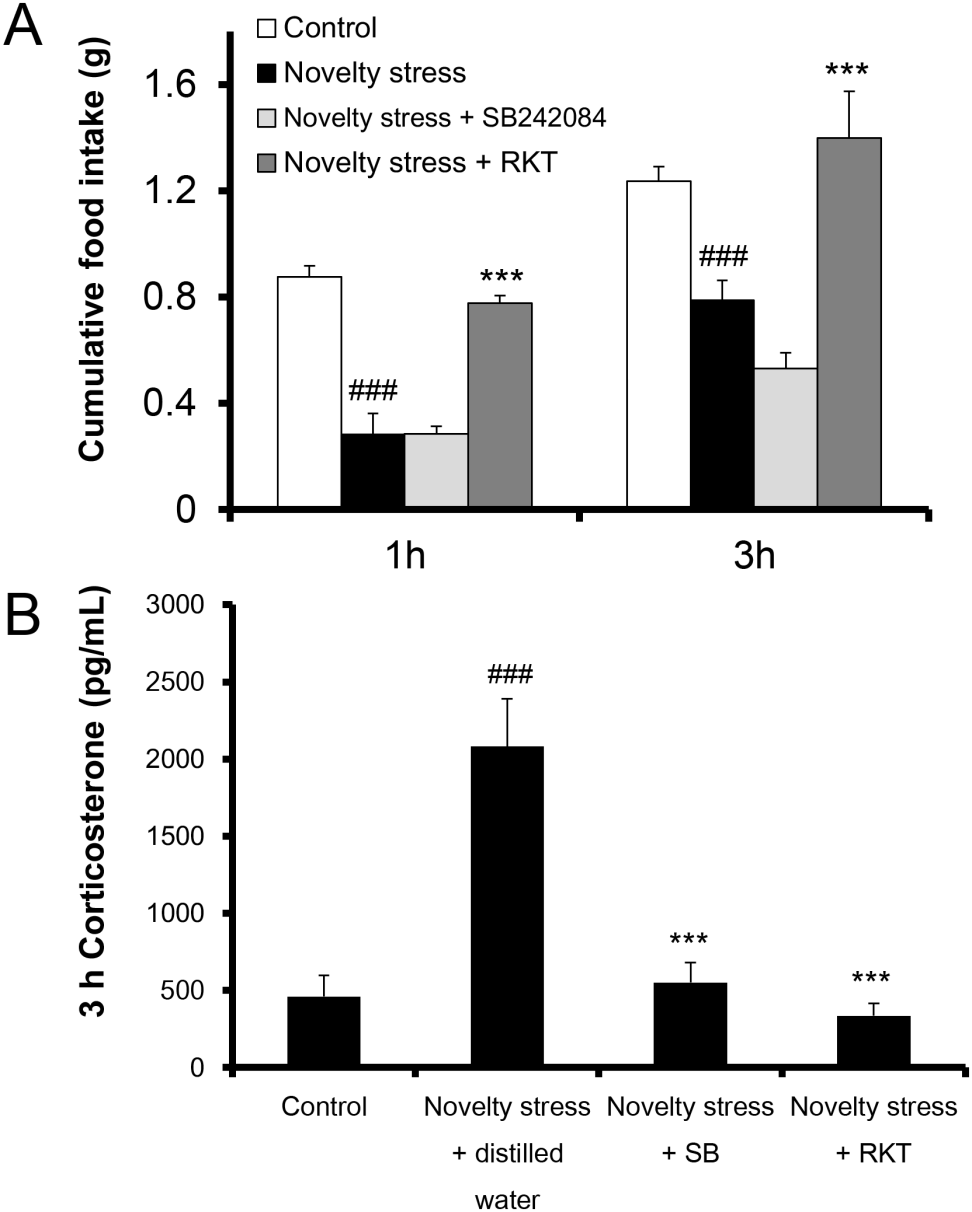
Effects of the 5-HT_2C_R antagonist and the endogenous ghrelin enhancer on cumulative food intake and the plasma corticosterone level in aged female mice exposed to novelty stress. 5-HT_2C_R antagonists, SB242084 (6 mg/kg, PO) or RKT(1000 mg/kg, PO) was administered immediately after stress exposure. (A) Cumulative food intake (1 h and 3 h) and (B) plasma corticosterone levels (3 h) after stress exposure was determined in 18 h fasted aged female mice. Data are presented as the mean ± SEM (n = 5–10). ###, p < 0.001 vs. control group, ***, p < 0.001 vs. novelty stress group.

Stress exposure to young female mice caused a significant decrease in food intake after 1 h [14], but no difference was found after 3 h (control; 0.90 ± 0.07 g, stress; 0.72 ± 0.08 g, p = 0.138, t-test, n = 5). At 1 h and 3 h after exposure to stress (acute phase) aged female mice had decreased food intakes (Fig 1A). The cumulative food intake in mice treated with SB242084 (6 mg/kg) was not restored to the control level; however, administration of RKT (1,000 mg/kg) to aged female mice exposed to stress significantly inhibited their decreased cumulative food intake (Fig 1A). The plasma corticosterone levels in aged female mice significantly increased at 3 h after stress exposure, and the administration of SB242084 and RKT significantly reduced the plasma corticosterone levels (Fig 1B).

### Changes in the plasma ghrelin levels of aged female mice exposed to novelty stress

**Fig 2:**
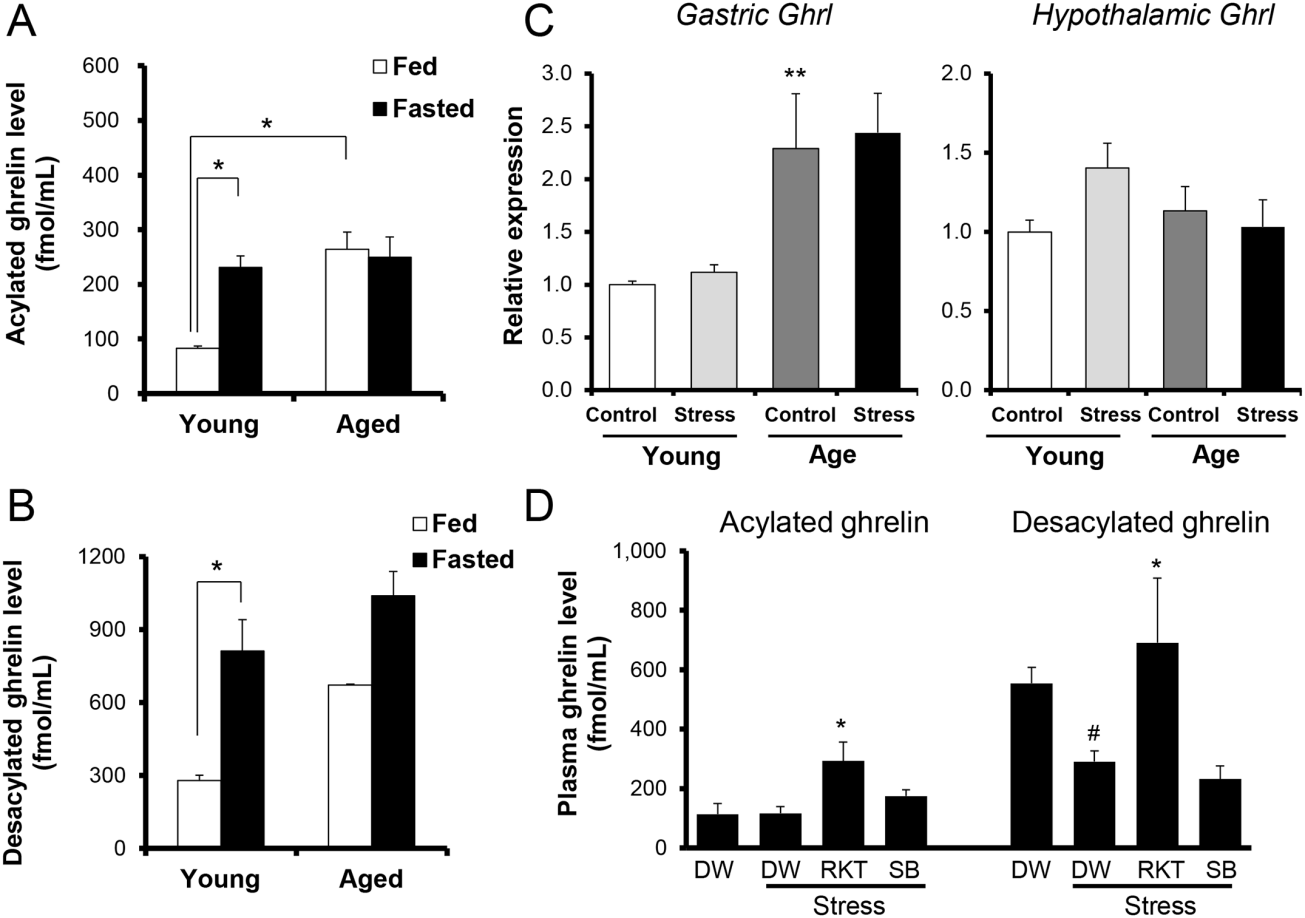
Changes in plasma ghrelin levels and preproghrelin mRNA expression in aged female mice exposed to novelty stress. Changes in the plasma levels of acylated ghrelin (A), desacylated ghrelin (B), gastric (left panel) and hypothalamic (right panel) preproghrelin mRNA expression (C) in the free-fed condition and the effects of a 5-HT_2C_R antagonist and endogenous ghrelin enhancer on plasma ghrelin levels in aged female mice exposed to novelty stress or not after an 18 h fast (D). The 5-HT_2C_R antagonist (SB242084; 6 mg/kg, PO), or ghrelin enhancer (rikkunshito (RKT); 1000 mg/kg, PO) was administered to aged female mice immediately after stress exposure. Data are presented as the mean ± SEM, (A, B) young; fed n = 9, fasted n = 8, aged; fed n = 3, fasted n = 5, (C) young; n = 8, aged; n = 5, (D) n = 5. (A-C); *, p < 0.05, **, p < 0.01 vs. young control group, (D); #, p < 0.05 vs. distilled water (DW)-treated non-stressed group. *, p < 0.05 vs. DW-treated stressed group.

In young female mice, plasma acylated and desacylated ghrelin levels (Fig 2A and 2B) were significantly increased by 18 h fasting. In contrast, both plasma ghrelin concentrations in aged female mice were not increased by 18 h fasting (Fig 2A and 2B). Plasma levels of acylated ghrelin in freely fed aged mice were higher than that in young mice, although the desacylated ghrelin concentration remained unchanged (Fig 2A and 2B). Compared with young mice, the expression of gastric preproghrelin mRNA in aged mice significantly increased; however, the increased expression of the hypothalamic gene was not identified after the exposure to stress (Fig 2C). The desacylated ghrelin level was significantly decreased after stress exposure, although the acylated ghrelin level remained unchanged (Fig 2D). Plasma ghrelin was significantly increased 3 h after exposure to stress by administration of RKT. SB242084 did not alter plasma ghrelin concentrations (Fig 2D).

**Fig 3:**
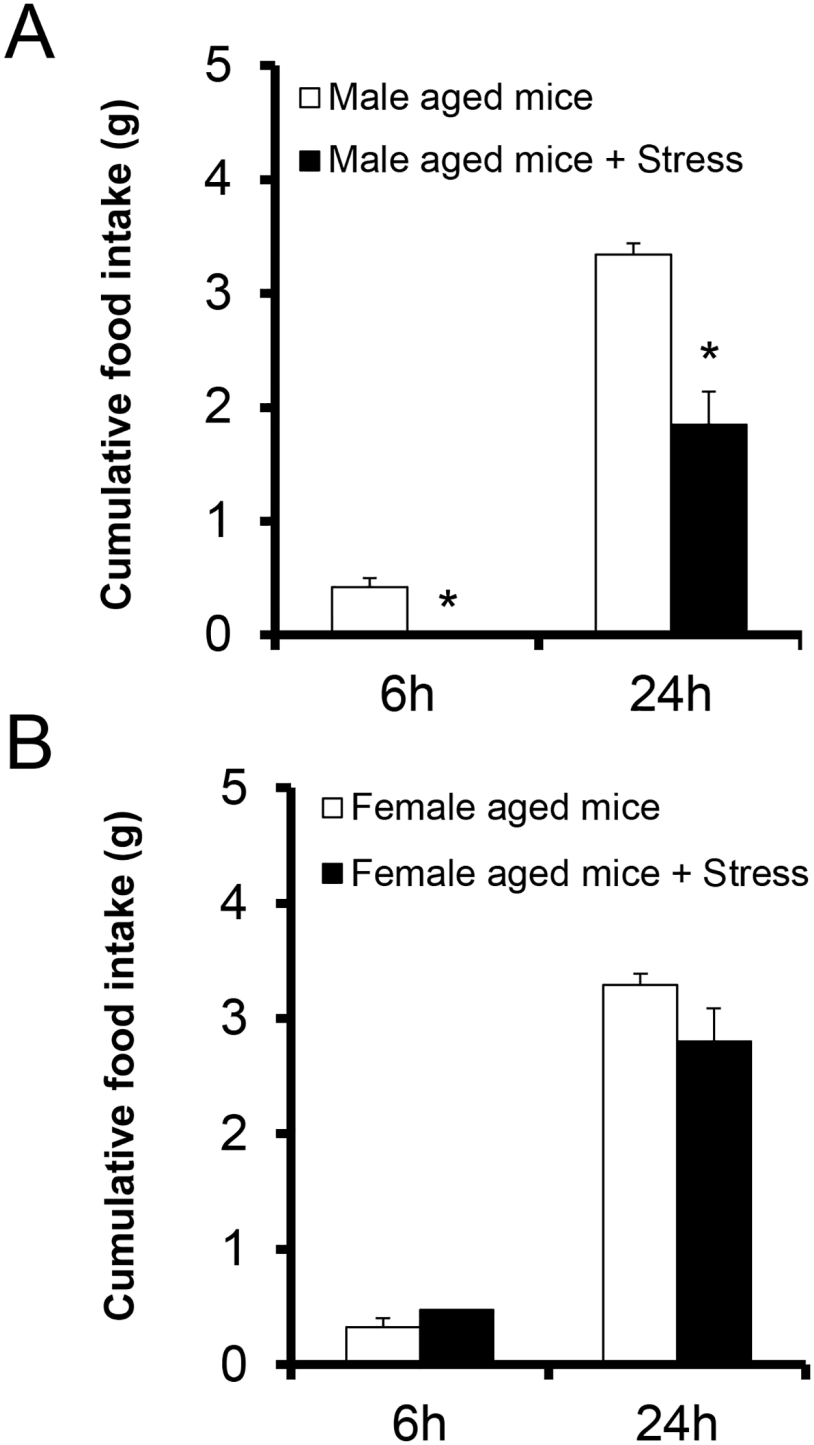
Changes in the cumulative food intake in aged male or female mice exposed to novelty stress. The cumulative food intake in aged male mice (A) and female mice (B) exposed to novelty stress during the fed condition. Data are presented as the mean ± SEM (n = 5). *, p < 0.05 vs. non-stressed group.

The food consumption in aged control and stressed male mice at 1 h was almost zero and in the stress group, the food consumption at 3 h was also remained zero (control; 0.27 ± 0.08 g, stress; 0.01 ± 0.01 g, p = 0.0286, t-test, n = 5). The food intake at 6 h or 24 h (late phase) in the aged male mice exposed to novelty stress decreased significantly compared with that of the control male aged mice (Fig 3A). The food intake of aged and stressed female mice did not decrease (Fig 3B).

### Changes in hypothalamic feeding-related gene and 5-HT_2C_R pre-RNA editing expression levels in young and old female mice

**Fig 4:**
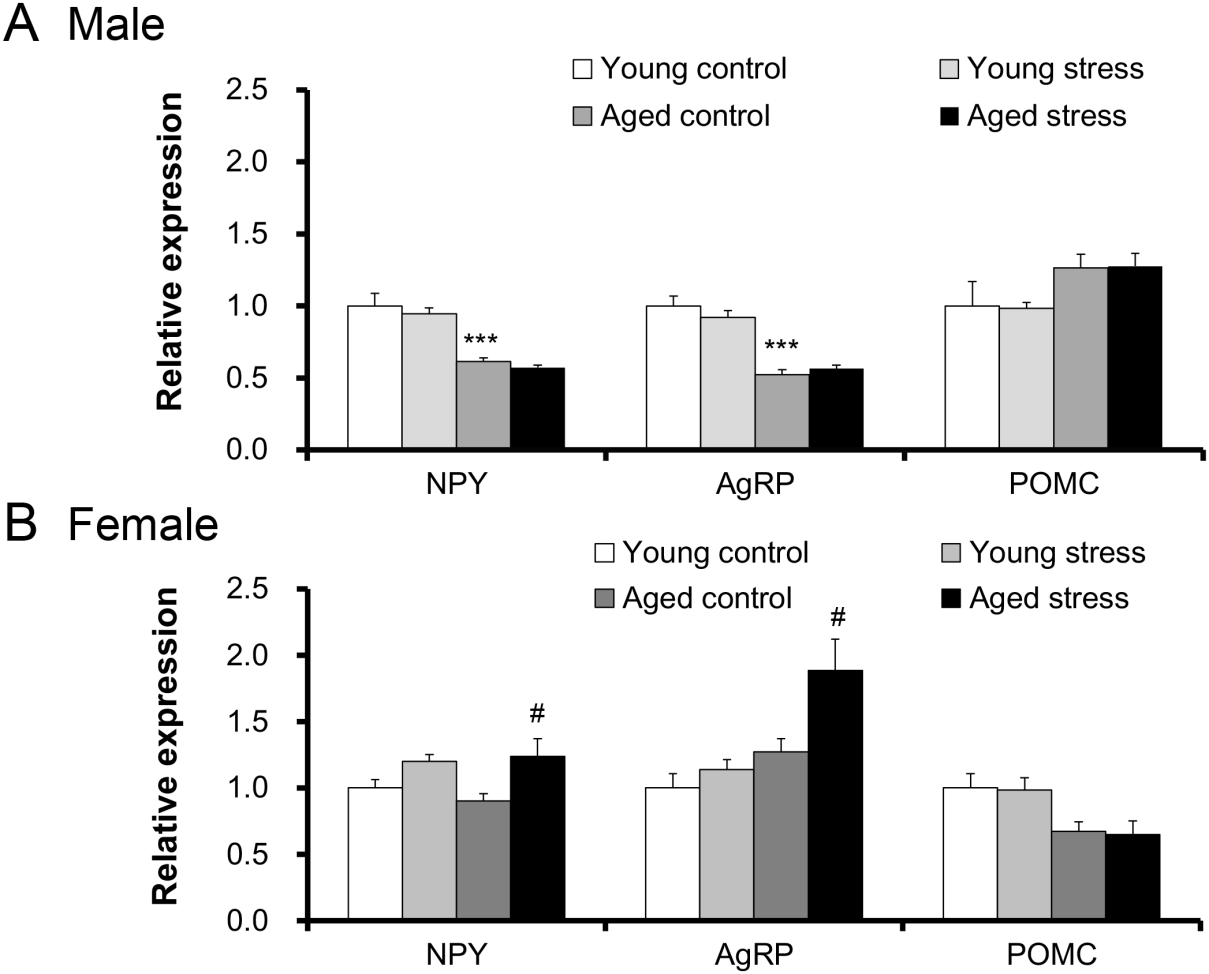
Changes in hypothalamic appetite-related peptide gene expression in aged male or female mice exposed to novelty stress. The 6 h hypothalamic appetite-related peptide in young and aged male mice (A), or female mice (B) exposed to novelty stress. Data are presented as the mean ± SEM (young male and aged female groups; n = 8, aged control female group; n = 7). ***, p < 0.001 vs. young control group. #, p < 0.05 vs. aged control group.

**Fig 5:**
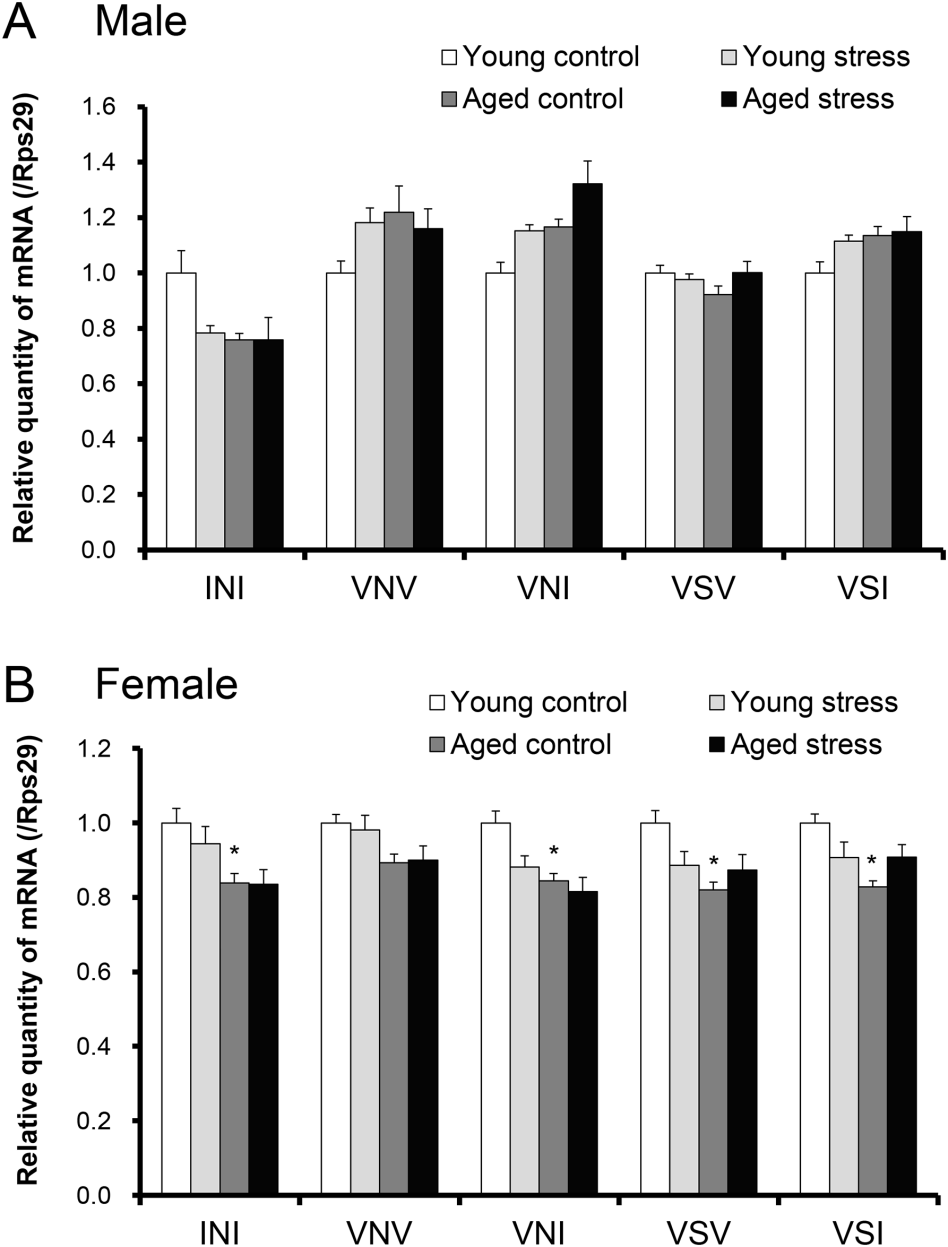
Changes in hypothalamic 5-HT_2C_R pre-RNA editing in aged male or female mice exposed to novelty stress. (A) Male mice, (B) Female mice. The hypothalami were harvested 6 h after stress exposure in 18 h-fasted mice. Data are presented as the mean ± SEM (young and aged stressed group; n = 8, aged male control group; n = 5, aged female control group; n = 7). *, p < 0.05 vs. young control group.

To clarify the mechanisms of mild reduction in food intake in aged female mice exposed to novelty stress, we focused on the pre-RNA editing expression levels of hypothalamic 5-HT_2C_R. First, we investigated the changes in the hypothalamic feeding-related gene in male and female mice (Fig 4A and 4B). In aged male mice, the mRNA expression of hypothalamic neuropeptide Y (NPY) or agouti-related protein (AgRP) significantly decreased compared with the expression levels in the young mice. In contrast, the expression levels of NPY and AgRP were significantly increased in stressed, aged female mice. Proopiomelanocortin (POMC) expression did not change in any of the groups. The hypothalamic expression of 5-HT_2C_R after exposure to stress did not change significantly changed in young mice (Fig 5A and 5B). The expression of the edited forms of 5-HT_2C_R (INI, VNI, VSV, VSI) were significantly decreased in aged female mice compared with young female mice (Fig 5B). However, 5-HT_2C_R gene expression did not change in aged female mice exposed to novelty stress (Fig 5B). The 5-HT_2C_R antagonists SB242084 and RKT failed to change the expression of the 5-HT_2C_R pre-RNA (data not shown).

### Changes in the number of ERα-positive cells in young and aged mice exposed to stress

**Fig 6:**
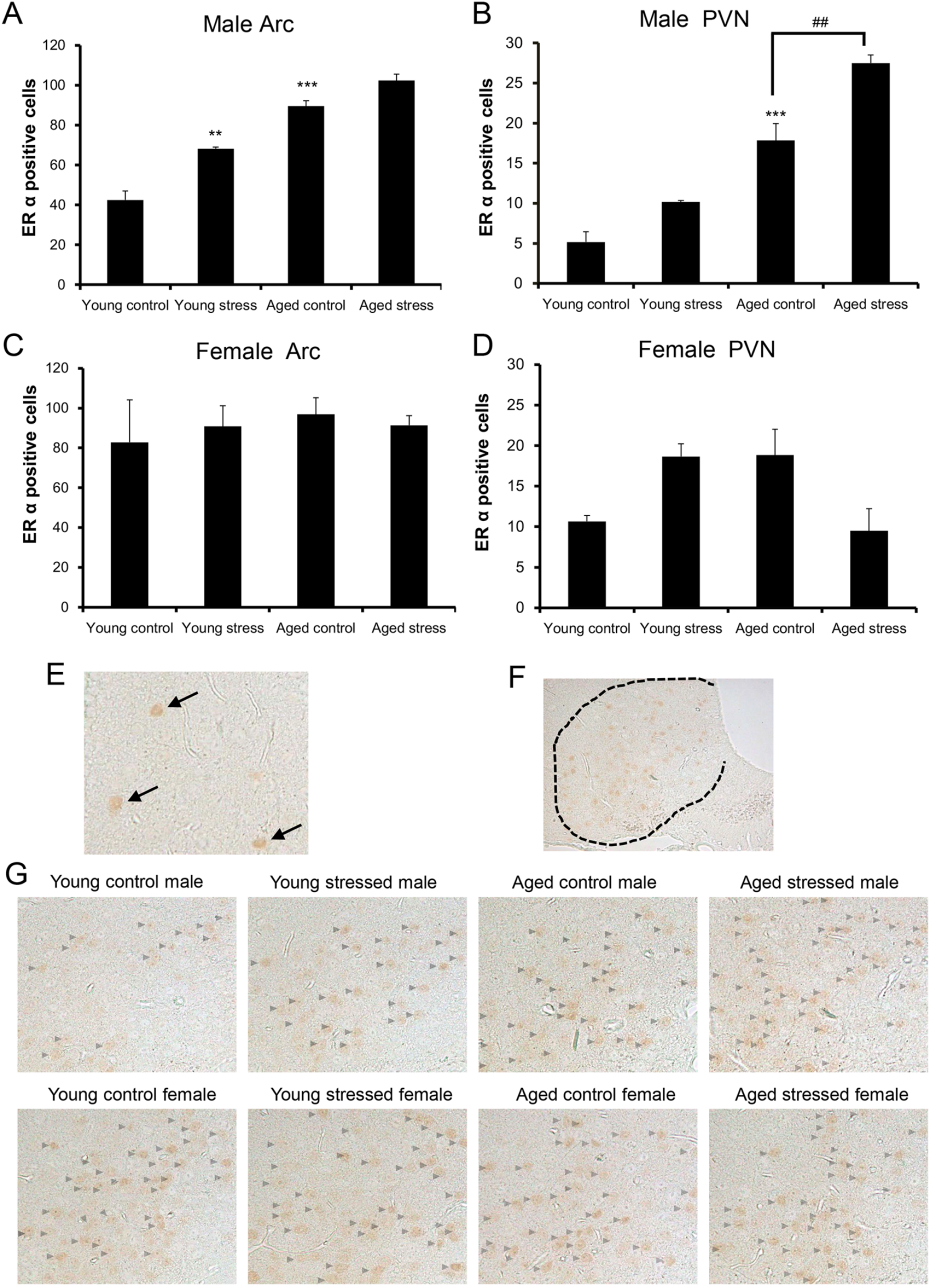
Changes in the number of ERα-positive cells in aged male or female mice exposed to novelty stress. (A) Male Arc, (B) Male PVN, (C) Female Arc, (D) Female PVN. The hypothalami were harvested from 18 h-fasted mice at 6 h after stress exposure. Data are presented as the mean ± SEM (young control, young stress, aged control groups; n = 3, aged stress group; n = 4). **, ***, p < 0.01, 0.001 vs. young control group. ##, p < 0.01 vs. aged control group. (E) Typical immunoreactivity for ERα-positive cells, (F) Measured Arc area, (G) ERα expression in the Arc.

Because ERα agonist-induced hypophagia in aged mice was clearly reversed by the administration of a 5-HT_2C_R antagonist [14], we determined the ERα-positive cell counts in the arcuate nucleus (Arc) and PVN in this study. Consistent with previous studies, ERα expression in the hypothalamus was localized in the nucleus [29]. As shown in Fig 6A, the number of ERα-positive cells in the Arc of young male mice was significantly increased by exposure to stress. A significant increase in the number of cells in the Arc and PVN (Fig 6A and 6B) in aged male control mice was observed compared to that in young control mice. The number of ERα-positive cells in the PVNs of aged male mice following stress exposure was significantly increased. Female mice did not show any changes in the number of ERα-positive cells in the Arc and PVN (Fig 6C and 6D). Typical cells showing immunoreactivity for ERα are indicated in Fig 6E, 6F and 6G.

## Discussion

This study is the first to find that (1) aged female mice showed an acute decrease in food intake after stress exposure, independent of the activation of the 5-HT_2C_R; (2) the administration of the endogenous ghrelin enhancer RKT significantly inhibited the decrease in food intake and the increase of plasma stress hormone; (3) a decrease in acylated ghrelin secretion in mice in the freely-fed condition following stress exposure was not observed in aged female mice; and (4) in aged female mice, a significant decrease in the expression of several hypothalamic 5-HT_2C_R editing isoforms and no changes in ERα expression in the hypothalamus were confirmed in comparison with young mice.

The cumulative food intake in aged mice exposed to stress clearly showed sex differences. In aged male mice, there was a sustained decrease in food intake until 24 h; however, in aged female mice, the decrease ended at 6 h. Similar results are apparent in a previous report [14]. The acute decrease in food intake in aged male mice exposed to stress is completely restored by the administration of a 5-HT_2C_R antagonist [14, 26]. In this study, however, an acute reduction in food intake in aged female mice was found and it was not reversed by the administration of the 5-HT_2C_R antagonist SB242084 (Fig 1A). This suggests that the reduction in feeding in aged female mice may not be mediated by the activation of 5-HT_2C_R. Unlike food consumption, the increased peripheral corticosterone level was completely suppressed by the administration of SB242084. Thus, the activation of the HPA axis may be involved in the activation of 5-HT_2C_R. A decrease in food intake in aged female mice may be caused by an alternative pathway to that of 5-HT_2C_R activation.

The secretion of peripheral ghrelin in young mice decreases during satiation and increases during fasting. This is due to increased ghrelin secretion by the X/A-like cells in the gastric mucosa during fasting, and the peripheral hunger signal is transferred to the central nervous system. The base peripheral concentration value of ghrelin in aged female mice was higher than that of young mice, and increased ghrelin secretion due to fasting was not been observed. It is likely that aging causes an abnormality in the transmission of the peripheral hunger signal in female mice. There are a few reports showing that hypophagia does not depend on the blood concentration of ghrelin or feeding behavior [42, 43]. Patients with anorexia nervosa (AN) who exhibit abnormalities in ghrelin responses and secretion experience a loss of appetite accompanied by high peripheral ghrelin concentrations [44]. Aged female mice may have an abnormality in ghrelin secretion that is similar to that of AN patients. To clarify the regulatory mechanisms of abnormal ghrelin secretion, we examined ghrelin synthesis in the stomachs of aged female mice. Since gastric preproghrelin gene expression increased during satiation in aged female mice compared with young mice, the high peripheral ghrelin levels evident during satiation might be due to the increased synthesis of ghrelin in the gastric mucosa. Activation of the 5-HT_2C_R reduces peripheral ghrelin secretion [32]. High basal ghrelin levels in aged female mice are likely to be due to reduced function of the 5-HT_2C_R. However, further studies are needed to determine whether hyperghrelinemia in aged female control mice is due to ghrelin deficiency.

Administration of the endogenous ghrelin enhancer, RKT, increases ghrelin binding to the growth hormone secretagogue receptor and enhances ghrelin secretion, activating the ghrelin signal [32, 33]. In this study, administration of RKT, but not the 5-HT_2C_R antagonist, to aged female mice exposed to novelty stress significantly increased their peripheral ghrelin levels. Thus, it may be necessary to stimulate ghrelin secretion to a more normal level to restore feeding to a normal level. Further research, such as direct administration of acylated ghrelin to aged female mice, is necessary to clarify ghrelin insufficiency in aged female mice.

The hypothalamus mediates peripheral nutritional status signals. Lower NPY/AgRP gene expression in aged male mice implies a blockade of peripheral hunger signals such as ghrelin signaling to the Arc. In female mice, these genes were clearly enhanced by stress loading. This result suggests that stress affects hunger stimuli in aged mice. The 5-HT_2C_R is expressed on POMC neurons, which stimulate the anorexic system, leading to the negative regulation of the activation of the orexigenic system NPY/AgRP neurons [45]. Since no further increase in peripheral ghrelin levels was observed in aged female mice after stress exposure, a significant increase in NPY/AgRP after stress exposure was thought to be caused by a peripheral fasting signal pathway other than ghrelin. Administration of RKT and SB242084 to aged male mice increases NPY/AgRP gene expression in the hypothalamus (S1 Fig), clearly inhibits hypophagia and decreases plasma ghrelin levels [26]. These changes were not observed in aged female mice in this study. Sex differences in NPY/AgRP gene expression in aged mice may be mediated by functional differences of 5-HT_2C_R.

Prolonged, reduced food intake in aged male mice following exposure to stress is caused by decreased peripheral ghrelin secretion dependent on activation of the 5-HT_2C_R [14]. To investigate the relationship between stress-induced, mild hypophagia in aged female mice and the 5-HT_2C_R, (1) the influence of serotonin secretion and/or the signal transduction factor of 5-HT_2C_R [46], (2) the intracerebral biosynthesis [47], and signaling [48, 49] has been examined so far. These findings may partly explain the 5-HT_2C_R dysfunction in aged mice exposed to stress.

In addition, the 5-HT_2C_R is the only 5-HTR with isoforms created by pre-mRNA editing. Changes in pre-mRNA editing with age may be responsible for the functional changes in 5-HT_2C_R. We also demonstrated that hypothalamic 5-HT_2C_R gene expression clearly increased in aged male mice exposed to stress compared with young male mice in previous studies [14, 16] and aimed to verify detailed gene expression profiles. We first examined the influence of aging on 5-HT_2C_R pre-mRNA editing in aged female mice.

The rate of editing differs by animal species and the brain area. The biochemical and pharmacological properties of 5-HT_2C_R are different and dependent on the rate of editing; the functional intensity of 5-HT_2C_R is highest in INI, the non-editing form, while the partial editing isoforms (VNV, VNI, VSV, VSI) reduce the functional intensity further. Regarding sex differences in5-HT_2C_R reactivity in stressed, aged mice, we hypothesized that the change of a specific pre-RNA editing isoform is partly involved. To determine if there was an altered 5-HT_2C_R editing profile within the hypophagic aged mice following exposure to stress, we investigated the gene expression of the pre-editing isoform of 5-HT_2C_R that is highly expressed in mouse strains [41]. Contrary to our expectation, the pre-editing 5-HT_2C_R mRNA expression in aged male mice exposed to stress was unchanged. In contrast, in female mice, the gene expression of INI, VNI, VSV, and VSI was significantly suppressed by age. Studies related to 5-HT_2C_R editing isoform expression and feeding behavior are extremely scarce. In this study, the proportion of each editing form was not verified. Suicidal patients with mood orders have decreased VNI gene expression [27]. Conversely, the VNI or VSV isoforms are significantly increased in ob/ob obese mice that exhibit hyperphagia [41]. The acute hypophagia in aged female mice may be partly due to the decreased expression of the VNI or VSV isoforms. In addition, we speculate that synthesis of the 5-HT_2C_R of isoforms, such as INI, in aged female mice was inhibited by age, and exposure of these mice to stress decreased synthesis of the highly reactive 5-HT_2C_R protein in the Arc, causing 5-HT_2C_R-independent hypophagia.

ERα activates the HPA in the hypothalamus PVN [50] and hypothalamic ERα gene expression is increased in aged male mice [14]. In addition, a 5-HT_2C_R antagonist reverses the ERα-induced hypophagia in age male mice [14]. The expression of ERα in aged male control mice was increased compared with that in young male mice. This result is consistent with that of a previous report [29] and suggested the possibility of hyper reactivity of central ERα in the aged male mice. Stress exposure in aged male mice clearly affected the expression of ERα in the PVN or the Arc (p = 0.0659); in contrast, aged female mice showed no change or tendency toward a similar decrease. Rather, sex differences in the sensitivity to hypophagia by ERα agonist administration in aged mice [14] may be due to differences in ERα expression. Thus, there is a possibility that the hyperactivity of 5-HT_2C_R promotes the protein synthesis of ERα.

## Conclusions

In summary, hypophagia in aged female mice exposed to stress may be independent of 5-HT_2C_R activation. It is likely that the mechanisms may be caused by sex-dependent, differential regulation of 5-HT_2C_R mRNA expression, peripheral acylated ghrelin secretion and/or hypothalamic ERα expression, as seen in S2 Fig.

## Supporting information

S1 Fig Effects of RKT or SB242048 on NPY, AgRP, POMC or CRF mRNA expression in the hypothalamus. The hypothalami were harvested from 18-h fasted mice at 6 h after exposure to stress. Data are presented as the mean ± SEM (n = 7–8). *, **, p < 0.05, 0.01 vs. stress group. NPY; neuropeptide Y, AgRP; agouti-related peptide, POMC; Proopiomelanocortin, CRF; corticotropin-releasing factor.
(TIF)

S2 Fig Proposed model for sex-dependent regulation of feeding behavior in aged mice under stress. In aged male mice, as already reported [1, 2], novelty stress decreases peripheral ghrelin secretion caused by elevated 5-HT_2C_R synthesis. These mechanisms mediate sustained suppression of food intake. Compared with young female mice, basal-aged female mice have decreased synthesis of 5-HT_2C_R and increased secretion of ghrelin. Food intake in aged female mice after exposure to stress was not affected by synthesis of the 5- HT_2C_R, secretion of ghrelin or ERα-expressing cell counts. Differences in feeding behavior between male and female aged mice exposed to stress exist.
(TIF)
