# Supplementary figures and images for: Psychological stress in aged female mice causes acute hypophagia independent of central serotonin 2C receptor activation
Source: PLoS One. 2017 Nov 10;12(11):e0187937. doi: 10.1371/journal.pone.0187937 (PMC5695286; doi:10.1371/journal.pone.0187937)

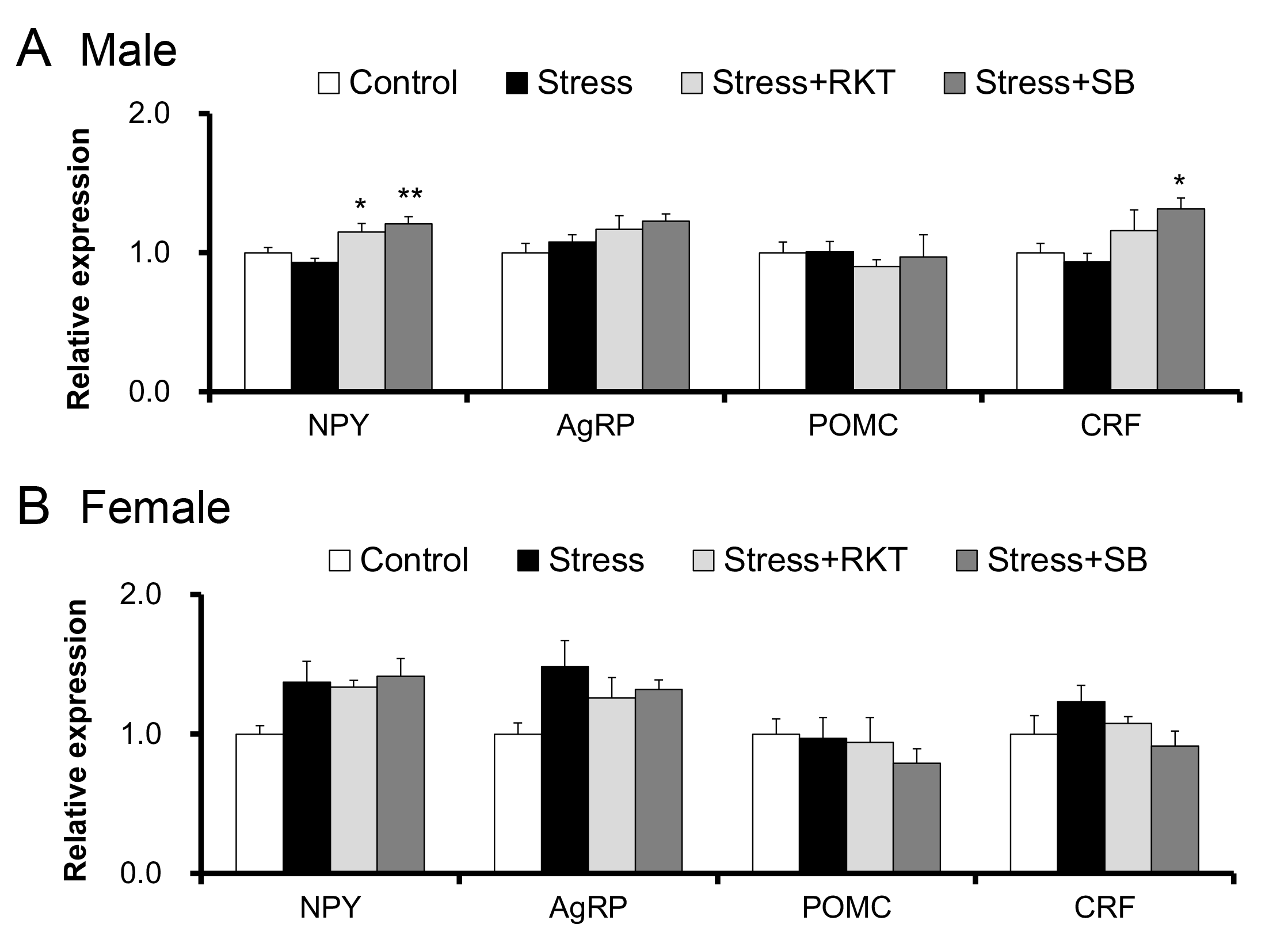

Supplement: S1 Fig — The hypothalami were harvested from 18-h fasted mice at 6 h after exposure to stress. Data are presented as the mean ± SEM (n = 7–8). *, **, p < 0.05, 0.01 vs. stress group. NPY; neuropeptide Y, AgRP; agouti-related peptide, POMC; Proopiomelanocortin, CRF; corticotropin-releasing factor. (TIF) [file pone.0187937.s001.tif]

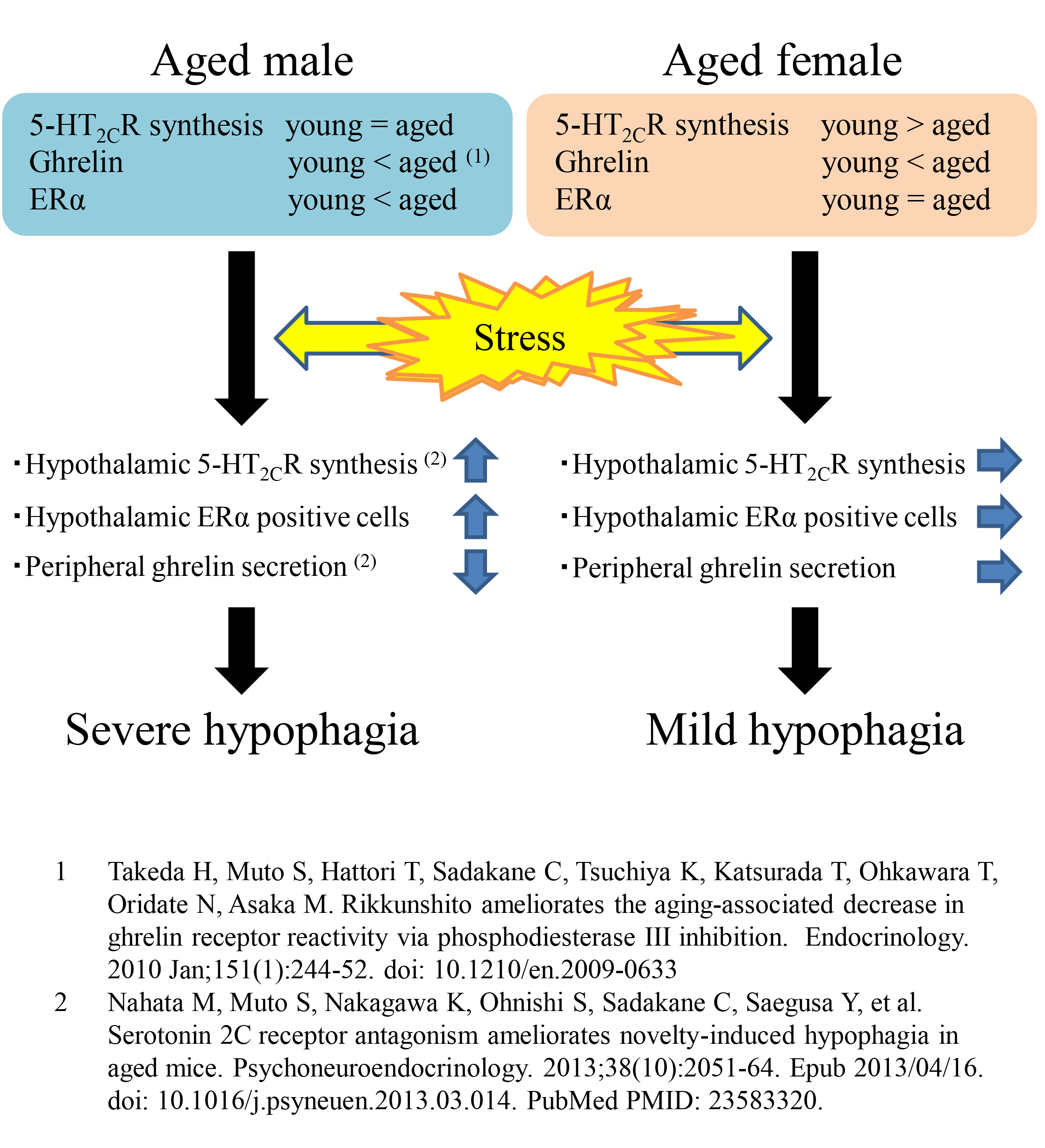

Supplement: S2 Fig — In aged male mice, as already reported [1, 2], novelty stress decreases peripheral ghrelin secretion caused by elevated 5-HT2CR synthesis. These mechanisms mediate sustained suppression of food intake. Compared with young female mice, basal-aged female mice have decreased synthesis of 5-HT2CR and increased secretion of ghrelin. Food intake in aged female mice after exposure to stress was not affected by synthesis of the 5- HT2CR, secretion of ghrelin or ERα-expressing cell counts. Differences in feeding behavior between male and female aged mice exposed to stress exist. (TIF) [file pone.0187937.s002.tif]
